# Sutured Intrascleral Posterior Chamber Intraocular Lens Fixation with Ciliary Sulcus Location Guided by Ultrasonic Biological Microscopy: A Retrospective Analysis of Anatomical and Refractive Outcome

**DOI:** 10.1155/2020/5843410

**Published:** 2020-06-04

**Authors:** Ya Qu, Ping Duan, Shujia Huo, Fuliang Li, Jiawen Li

**Affiliations:** Southwest Hospital/Southwest Eye Hospital, Third Military Medical University (Army Medical University), 30th Gaotanyan Street, Shapingba, Chongqing 400038, China

## Abstract

**Purpose:**

To report the outcome of sutured intrascleral posterior chamber intraocular lens (PC IOL) fixation with ciliary sulcus location guided by ultrasonic biological microscopy (UBM).

**Methods:**

Patients who underwent a sutured intrascleral PC IOL fixation were reviewed and divided into four groups. In group 1, the traditional sulcus fixation (2 mm from limbus) of IOL was performed. In groups 2, 3, and 4, UBM was performed before surgery to locate the position of the ciliary sulcus as the haptics insertion position. IOL power was selected by decreasing the calculated value of the IOL power by 1.0 D, 1.0 D, 0.5 D, and 0.0 D, respectively.

**Results:**

Sixty-one patients (63 eyes) were included in the four groups. After 4.1 ± 3.0 months' follow-up, the postsurgery spherical equivalent (SE) was 0.73 ± 1.86, 0.71 ± 0.84, 1.14 ± 0.45, and 0.07 ± 0.89 diopters (D), respectively. Statistical significance was reached for the postsurgery SE with target refraction between group 1 (*p* = 0.027, <0.05), group 2 (*p* = 0.003, <0.01), and group 3 (*p* = 0.017, <0.05). No significant difference existed for the postsurgery SE with target refraction in group 4 (*p* = 0.779, >0.05), and the postsurgery SE in group 4 was the nearest to target refraction.

**Conclusion:**

Intrascleral PC IOL fixation guided by UBM is helpful for locating the ciliary sulcus and satisfactory visual outcomes with a predictable IOL power calculation.

## 1. Introduction

Implantation of an intraocular lens (IOL) in an eye without sufficient capsule support after vitrectomy can be accomplished by using an anterior chamber IOL, an iris-fixated IOL, a sutured or sutureless transscleral-fixated posterior chamber IOL (PC IOL), and transconjunctival intrascleral IOL [1, 2]. The sutureless intrascleral fixation technique of PC IOL has been the subject of a number of recent reports, and it is capable of obtaining a satisfactory effect [3–6]. Here, we present our initial experience with the sutured intrascleral fixation technique of PC IOL under the guidance of ultrasonic biological microscopuy (UBM) involved in the ciliary sulcus location.

## 2. Materials and Methods

### 2.1. Subjects

This study is retrospective. 61 patients (63 eyes), including 53 men (55 eyes) and 8 women (8 eyes), with a mean age of 48.6 ± 14.9 years (range 23 to 85 years) from June 2015 to May 2016 in our hospital were collected.  Table 1 shows the baseline characteristics and preexisting ocular conditions of the patients.

Inclusion criteria include the following: (1) the patients with aphakia underwent 23-/25-gauge vitrectomy or anterior vitrectomy because of eye trauma, retinal detachment, lens luxation, or Marfan syndrome, and the IOL was impossible to implant at the first procedure; (2) a three-piece foldable IOL was implanted into these aphakic eyes as a final procedure 2-3 months from the last procedure; (3) age ≥18 years; and (4) the best-corrected visual acuity (BCVA) ≥ 0.1 (logMAR 1.0). Exclusion criteria include the following: (1) eyes with axial lengths <22 mm and ≥30 mm; (2) follow-up time less than 1 month. All procedures were in accordance with the ethical standards of the Declaration of Helsinki.

All patients underwent comprehensive ophthalmic evaluation, including uncorrected visual acuity (UCVA), best-corrected visual acuity (BCVA), noncontact tonometry (NCT), slit-lamp examination (SLE), corneal endothelial cell density, IOL master measure, B scan, UBM, and macular optical coherence tomography.

### 2.2. Examination Using an Ultrasonic Biological Microscope

UBM (SW-3200, KINSCAN, SUOER) was performed for 43 patients (44 eyes) to locate the ciliary sulcus before surgery [7], and the distance from the corneal limbus to ciliary sulcus on the scleral surface at the 1 o'clock and 7 o'clock positions was recorded as the point of IOL haptic insertion.

### 2.3. Patient Grouping and Intraocular Lens Power Selection

The target refraction in all cases was –0.00 D. For the 18 patients (19 eyes) of IOL haptic insertion point designed 2 mm posterior to the limbus, the final power was selected by decreasing the calculated value of the IOL power by 1.0 D because of the slight anterior location of the sulcus compared with capsular bag placement (group 1). For the 43 patients (44 eyes) of IOL haptic insertion point designed as ciliary sulcus according to UBM, three other kinds of IOL power were selected by decreasing the calculated value of the IOL power by 1.0 D, 0.5 D, and 0.0 D, respectively (groups 2, 3, and 4), to reach the target refraction [8]. All IOL powers were calculated by SRK-T formula.

### 2.4. Surgical Procedure

**Figure 1:**
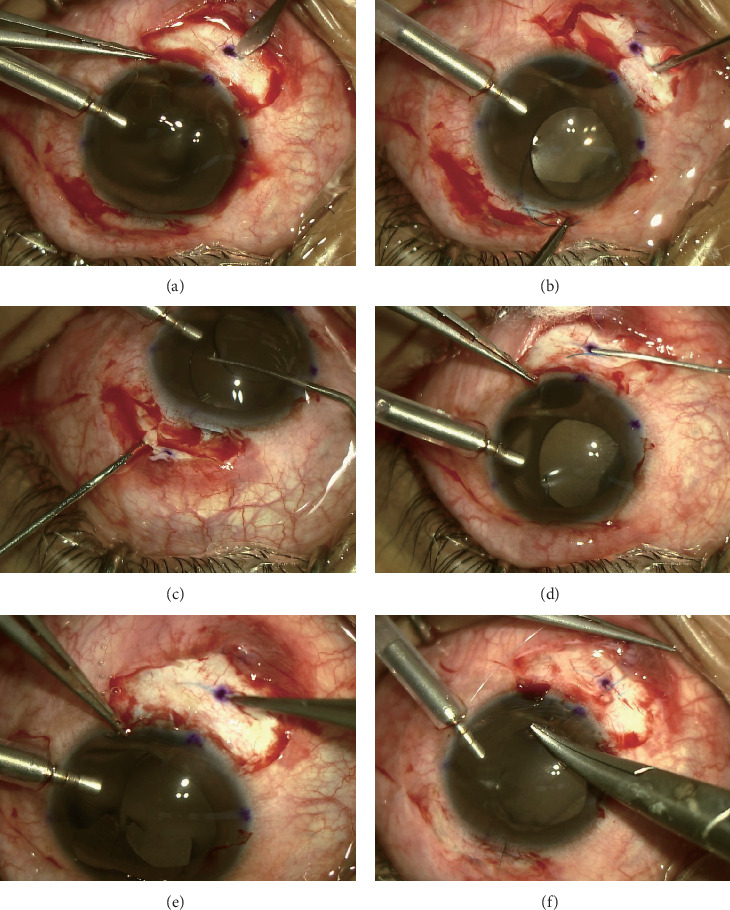
Photographs showing the sutured intrascleral posterior chamber intraocular lens fixation with ciliary sulcus location. (a) Two-millimeter scleral incisions were made at 1 o'clock and 7 o'clock. (b) The leading haptic of a three-piece IOL was held with forceps and then extracted from the eye through the 7 o'clock scleral incisions. (c) The trailing haptic was held with forceps and externalized onto the sclera through the 1 o'clock scleral incisions. (d) Two 3.0 mm scleral tunnels (1 o'clock and 7 o'clock) of approximately 30% scleral thickness were created along the limbus using a disposable syringe needle. (e) The haptics were inserted approximately 2-3 mm into the scleral tunnel. (f) The scleral incision was closed with 9–0 nonabsorbable sutures that were tied to fixate the haptic.

An anterior chamber maintainer was adopted to stabilize intraocular pressure (IOP) under retrobulbar anesthesia, and two 2 mm sclera incisions were made with a paracentesis blade at two points 180° apart at the 1 o'clock and 7 o'clock meridians according to the design (Figure 1(a)). A three-piece IOL (MA60AC, Alcon Laboratories, Inc.) was inserted into the anterior chamber, and the leading haptic was extracted from the eye through the 7 o'clock scleral incision with forceps (Grieshaber 23-gauge, Alcon Laboratories, Inc.) (Figure 1(b)); then, the trailing haptic was also externalized onto the sclera (Figure 1(c)). Two limbal parallel scleral tunnels besides the incisions were made using a disposable syringe needle (1 ml, outside diameter 0.7 mm) (Figure 1(d)); then, the haptics were inserted into them (Figure 1(e)). The scleral incision was closed with 9–0 nonabsorbable sutures (Polypropylene sutures, 1465P, MANI) to fixate the two haptics (Figure 1(f)). At the completion of surgery, the anterior chamber maintainer was removed, and all corneal incision and sclerotomy sites were inspected for wound leakage. Following surgery, UCVA, BCVA, NCT, SLE, UBM, or anterior segment OCT were recorded at follow-up.

### 2.5. Statistics

All the data were entered in Microsoft Excel sheets and analyzed using SPSS 21.0. Continuous variables were described as means ± standard deviation (*x* ± *s*). Visual acuity was converted to logarithm of minimum angle of resolution (logMAR) for analysis. Single-factor analysis of variance was used to examine whether a significant difference existed in the position of ciliary sulcus and in axial lengths between groups. Wilcoxon tests have been used for comparison between target refraction and spherical equivalent after surgery. A *p* value < 0.05 was considered statistically significant.

## 3. Results and Discussion

### 3.1. Results

UBM examination was performed on 43 patients (44 eyes) to locate the ciliary sulcus on sclera, and the distance from the corneal limbus to the ciliary sulcus at the 1 o'clock and 7 o'clock positions were recorded as the IOL haptic insertion points. The distance of ciliary sulcus on the sclera surface was 2.40 ± 0.26 mm at the 1 o'clock position and 2.35 ± 0.23 mm at the 7 o'clock position of the 44 eyes, and the average distance was 2.44 ± 0.24 mm, 2.41 ± 0.16 mm, and 2.27 ± 0.22 mm, respectively in groups 2, 3, and 4, respectively. A significant difference was not found in the position of ciliary sulcus between the three groups (*F* = 2.622, *p* > 0.05). The axial length of all eyes was 23.78 ± 1.18 mm and of eyes in each group was 24.02 ± 1.58 mm, 23.70 ± 1.03 mm, 23.92 ± 0.71 mm, and 23.53 ± 0.99 mm, respectively. There were no statistically significant differences between these groups in the axial lengths (*F* = 0.515, *p* > 0.05) (Table 2).

The UCVA and BCVA before and after surgery of all cases are listed in Table 2. After 4.1 ± 3.0 months' follow-up, the UCVA was 0.55 ± 0.30, 0.60 ± 0.34, 0.51 ± 0.37, and 0.47 ± 0.34 (logMAR), respectively. The mean spherical equivalent (SE) after surgery of traditional position implantation (group 1) was 0.73 D ± 1.86, and the postsurgery SE of ciliary sulcus implantation (groups 2, 3, and 4) was 0.71 D ± 0.84, 1.14 D ± 0.45, and 0.07 D ± 0.89, respectively. Statistical significance was reached for the postsurgery SE with target refraction between group 1 (*p*=0.027, <0.05), group 2 (*p*=0.003, <0.01), and group 3 (*p*=0.017, <0.05). No significant difference existed for the postsurgery SE with target refraction in group 4 (*p*=0.779, >0.05), and the postsurgery SE in group 4 was the nearest to the target refraction.

The postoperative complications included iris capture of the IOL in 1 eye (0.016%), vitreous hemorrhage in 2 eyes (0.032%), retinal detachment in 1 eye (0.016%), and transient ocular hypertension in 3 eyes (0.05%). The iris capture of the IOL was recovered after YAG laser iridectomy [9]. Transient ocular hypertension mostly occurred in the first week and was controlled well by gradual reduction in eye drops until complete withdrawal. The case of retinal detachment underwent vitrectomy next. For 1 eye of repeated vitreous hemorrhage accompanied by ocular hypertension (16–40 mmHg), intraocular lavage was performed, and finally, the IOL was taken out. No other complications, endophthalmitis, IOL dislocations, or haptic sliding from the tunnel were detected during the follow-up period.

### 3.2. Discussion

**Figure 2:**
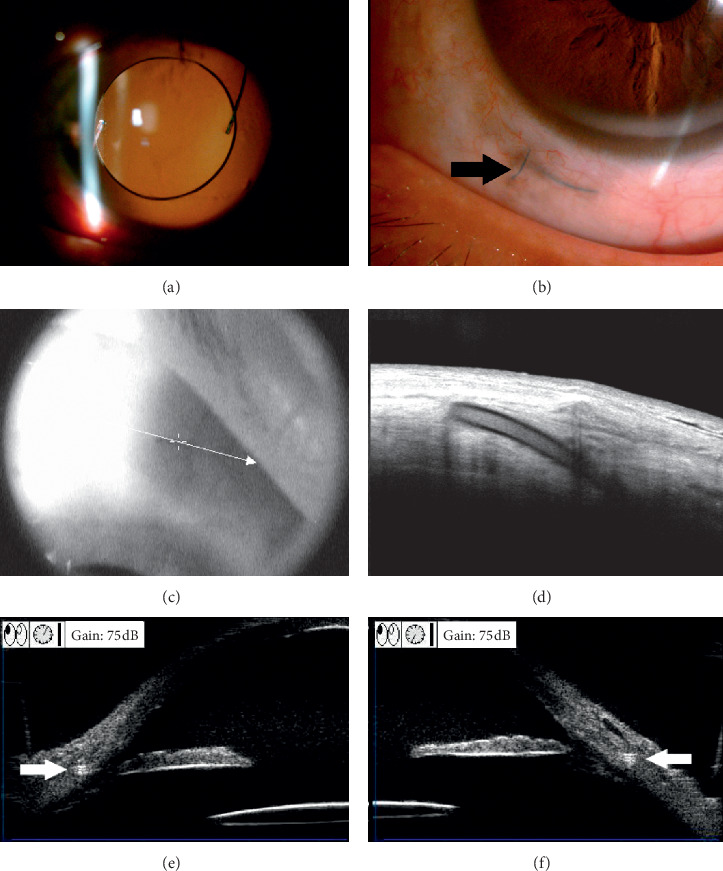
Anterior segment image of the eye after the sutured intrascleral PC IOL fixation surgery. (a) Photograph of a well-centered PC IOL three months after operation with a iridocoloboma caused by blunt trauma. (b) Three-month postoperative slit lamp image of the limbal position. The PC IOL haptic (black arrow) was completely incarcerated in the scleral tunnel. (c, d) Anterior segment OCT demonstrating intrascleral haptic of the PC IOL. (e, f) The UBM appearance of haptics of the IOL was in the sclera, which was characterized by high echoes during the UBM examination (white arrow).

Intrascleral fixation of PC IOL has become more popular, as it has advantages such as minimal trauma to the surrounding tissues, good IOL stabilization decreasing the incidence of IOL tilt, and shorter operation time, and it does not require degradable threads, which may lead to long-term extraconjunctival exposure (Figure 2) [10, 11].

With our technique, we modified the haptic externalization techniques with a 23 g-forceps because we found that it was difficult to insert the haptic into the needles for next externalization and the haptic onto the sclera at the posterior chamber or anterior vitreous cavity if without enough experience [9, 12, 13]. A small 2 mm incision of sclera is conducive to haptic externalization with force because the haptic is easily grasped and has no haptic distortion [1, 13]. The IOL haptic could be inserted into the scleral tunnel visually which was made with a disposable syringe needle and be fastened in the scleral tunnel.

From the prior viewpoint, implantation of a PC IOL in an eye without sufficient capsule support was mostly fixed at 1.5–2 mm from the limbus which is regarded as the position of ciliary sulcus [14, 15]. Refractive outcomes from sulcus-implanted PC IOLs are different from in-the-bag IOLs because the IOL moves anteriorly [16, 17], necessitating a reduction of 1.0 D or 0.5 D in the IOL power placed at the sulcus plane in order to achieve the same refractive effect [18, 19].

However, very few studies related to intrascleral fixation of PC IOL reported the postoperative refractive error. A mild myopic shift of −0.21 D ± 0.99 was reported by Yamane et al., whereas Czajka et al. showed that the mean postoperative shift was +0.19 D ± 1.05 [1, 20]. Kawaji et al. reported a mild hyperopic shift of +0.25 D ± 0.79 when the IOL haptic was placed 2.0 mm parallel to the limbus, and Abbey et al. reported that the mild hyperopic shift was +0.41 D for which IOL placement at 1.5 mm accounts for approximately 0.23 D of hyperopic shift away from the target SE when compared to IOL placement at 2 mm [21, 22]. Hyperopic shift seems a problem that was likely to be encountered. In our study, residual refractive outcomes accounts for a hyperopic shift of +0.73D ± 1.86 in the group of 2 mm from the limbus (Table 2, group 1), so a more accurate location of ciliary sulcus and the corresponding degree of intrasulcus fixation IOL were need to be predicted.

UBM is suitable for imaging of anterior segment anatomy and pathology and useful for assessment of anterior chamber depth, lens tilt, sulcus diameter, and haptics position in sulcus fixation of PC IOL [7, 23, 24]. So we intended to look for the accurate ciliary sulcus location as the physiological lens position for each case with UBM examination; in other words, the postoperation refraction was our target.

In group 2, the hyperopic shift still remained with this viewpoint of reduction of 1.0 D in the calculated IOL power, so IOL power was adjusted in time in later cases involved in group 3 (calculated value: −0.5D) and group 4 (calculated value: −0.0D) to avoid the hyperopic shift (Table 2). The UCVA of patients in the UBM-aided group was found better than that in the control group, which means the postsurgery UCVA was related to the suturing position. The postsurgery SE was minimal in group 4 such that IOL power selected was the same as that of intracapsular implantation, which indicates that UBM provides personalized positioning of the ciliary sulcus and reduces the deviation of the surgical suture caused by anatomic differences [7].

Vitreous hemorrhage was a common complication with relatively few incidences (2.5%–16.6%.); most of them were all self-resorbing within days [20, 25]. One exception reported intraocular hemorrhage in 13 out of 25 eyes (52%) in which the suture was placed 1.0 mm posterior to the limbus [26]. However, there is discussion about the location of the true sulcus space relative to the limbus [27, 28]. In our cases, there were relatively few incidences of vitreous hemorrhage (2/62, 0.032%), suggesting that allowing the lens to sit in the true sulcus space and avoiding the vascular of ciliary body may reduce the risk of bleeding [14, 29]. Repeated vitreous hemorrhage in 1 eye was in group 1 with the IOL haptic location not according to UBM and was related to the attendant risk of IOL haptic contact with vascular area of ciliary body, which was confirmed by vitreous hemorrhage no longer occurring after removing the IOL.

The problems of IOL dislocation and tilt still exist in some sutureless techniques, so the scleral incision and 2-3 mm scleral tunnel with IOL haptic should be sutured at the end of the surgery for tight fixation [3, 9]. Unavoidable mild decentering of PC IOL was managed with appropriate refractive correction. The haptic tips were inside the tunnel to prevent foreign-body sensation or conjunctival erosion and reduce inflammation-causing stimuli [30].

## 4. Conclusions

It can be concluded that UBM can help locate ciliary sulcus on sclera to be the accurate site of IOL haptic fixation, which is close to the physiological lens position. With our methods, refraction in eyes with sulcus-implanted IOLs can be predicted with the same accuracy as in eyes with in-the-bag IOLs. There are limitations to our study, including a small sample size and short follow-up period. Future studies are required to understand the long-term implications and stability of this surgical technique for accurate IOL position, including tilt and decentration of the IOL.

## Figures and Tables

**Table 1:** Baseline characteristics and preexisting conditions.

Characteristics	Value
Patients (eyes)	61 (63)
Males (eyes)	53 (55)
Females (eyes)	8 (8)
Mean age (*y*) ± SD	48.6 ± 14.9
Mean axial lengths (mm) ±S D	23.78 ± 1.18
Preexisting conditions	Patients (eyes)
Trauma (intraocular foreign body, penetrating wound, rupture wound)	46 (46)
Lens subluxation	7 (7)
Retina detachment	4 (4)
Zonulysis (high myopia)	3 (4)
Lens luxation by Marfan syndrome	1 (2)
Mean follow-up (mo) ± SD	4.1 ± 3.0
Mean baseline UCVA (logMAR)	1.72 ± 0.29
Mean baseline BCVA (logMAR)	0.34 ± 0.36
Mean baseline spherical equivalent (SE)	10.67 ± 2.85

UCVA = uncorrected visual acuity; BCVA = best-corrected visual acuity.

**Table 2:** Ciliary sulcus position and visual acuity.

Group	IOL haptic inserted point	IOL power selected (D)	Patients (eyes)	Axial lengths (mm)	The distance from corneal limbus to ciliary sulcus			Presurgery (logMAR)		Postsurgery (logMAR)		Postsurgery SE		
					1 o' clock	7 o' clock	Average	UCVA	BCVA	UCVA	BCVA	SE	*Z*	*p*
1	2 mm posterior to limbus	CA–1.0	18 (19)	24.02 ± 1.58	None	None	None	1.79 ± 0.27	0.41 ± 0.34	0.55 ± 0.30	0.32 ± 0.28	0.73 ± 1.86	2.216	0.027
2	Ciliary sulcus implantation by UBM	CA–1.0	23 (24)	23.70 ± 1.03	2.47 ± 0.26	2.39 ± 0.24	2.44 ± 0.24	1.67 ± 0.34	0.33 ± 0.21	0.60 ± 0.34	0.33 ± 0.24	0.71 ± 0.84	2.981	0.003
3		CA–0.5	7 (7)	23.92 ± 0.71	2.45 ± 0.16	2.37 ± 0.16	2.41 ± 0.16	1.56 ± 0.28	0.11 ± 0.56	0.51 ± 0.37	0.34 ± 0.37	1.14 ± 0.45	2.388	0.017
4		CA–0.0	13 (13)	23.53 ± 0.99	2.27 ± 0.26	2.26 ± 0.21	2.27 ± 0.22	1.77 ± 0.23	0.37 ± 0.42	0.47 ± 0.34	0.39 ± 0.34	0.07 ± 0.89	0.281	0.779

All			61 (63)	*F* = 0.515 *p*=0.085	2.40 ± 0.26	2.35 ± 0.23	*F* = 2.622 *p*=0.085	1.72 ± 0.29	0.34 ± 0.36	0.55 ± 0.33	0.34 ± 0.29	0.63 ± 1.24		

IOL = intraocular lens; CA = calculated value; D = diopters; logMAR = logarithm of the minimum angle of resolution; UCVA = uncorrected visual acuity; BCVA = best-corrected visual acuity; SE = spherical equivalent. Data are mean ± standard deviation.

## Data Availability

All data generated or analyzed during this study are included in this article.
